# Focal adhesion kinase and its epigenetic interactors as diagnostic and therapeutic hints for pediatric hepatoblastoma

**DOI:** 10.3389/fonc.2024.1397647

**Published:** 2024-06-14

**Authors:** Maria Rita Braghini, Cristiano De Stefanis, Francesca Tiano, Aurora Castellano, Nicolo’ Cicolani, Marco Pezzullo, Valeria Tocco, Marco Spada, Rita Alaggio, Anna Alisi, Paola Francalanci

**Affiliations:** ^1^ Research Unit of Genetics of Complex Phenotypes, Bambino Gesù Children’s Hospital, IRCCS, Rome, Italy; ^2^ Core Facilities, Bambino Gesù Children’s Hospital, IRCCS, Rome, Italy; ^3^ Division of Oncohematology, Bambino Gesù Children’s Hospital, IRCCS, Rome, Italy; ^4^ Research Unit of Clinical Hepatogastroenterology and Transplantation; Division of Hepatobiliopancreatic Surgery, Liver and Kidney Transplantation, Bambino Gesù Children’s Hospital, IRCCS, Rome, Italy; ^5^ Pathology Unit, Bambino Gesù Children’s Hospital, IRCCS, Rome, Italy

**Keywords:** hepatoblastoma, focal adhesion kinase, epigenetic regulators, therapy, tumor size

## Abstract

**Background:**

Hepatoblastoma (HB) is the most common pediatric hepatic malignancy. Despite the progress in HB treatment, investigating HB pathomechanisms to optimize stratification and therapies remains a focal point to improve the outcome for high-risk patients.

**Methods:**

Here, we pointed to explore the impact of these mechanisms in HB. An observational study was performed on liver samples from a cohort of 17 patients with a diagnosis of HB and two normal liver samples. The *in vitro* experiments were executed on the Huh6 human HB cell line treated with the FAK inhibitor TAE226.

**Results:**

Our results highlight a significant up-regulation of mRNA and protein expression of FAK in livers from HB with respect to normal livers. The increased protein expression of total and Tyr397 phosphorylated FAK (pTyr397FAK) was significantly correlated with the expression of some epigenetic regulators of histone H3 methylation and acetylation. Of note, the expression of pTyr397FAK, N-methyltransferase enzyme (EZH2) and tri-methylation of the H3K27 residue correlated with tumor size and alpha-fetoprotein (AFP) levels. Finally, TAE226 caused a significant reduction of pTyr397FAK, epigenetic regulators, *AFP*, *EPCAM*, *OCT4*, and *SOX2*, in association with anti-proliferative and pro-apoptotic effects on HB cells.

**Conclusion:**

Our results suggest a role of FAK in HB that requires further investigations mainly focused on the exploration of its effective diagnostic and therapeutic translatability.

## Introduction

Hepatoblastoma (HB) is the most common pediatric liver malignancy (1). During the last few years, interventional approaches against HB have progressed and currently include liver resection, neoadjuvant chemotherapy and liver transplantation. These therapeutic advancements have strongly contributed to the prolonged disease-free survival rate of patients with HB. Indeed, the disease-free survival rate at 5 years in low-risk children with HB has steadily increased from 27% reported in the 1990s to the current 80–90% (2–4). The improvement in survival rate in HB is ascribable to standardized risk stratification guidelines recently developed by an international consensus group called the Children’s Hepatic Tumors International Collaboration (CHIC) (5). This stratification is based on several features, such as the age of the patients, the serum levels of alpha-fetoprotein (AFP), the presence of metastatic disease, and other factors (i.e., vascular involvement, extrahepatic contiguous extension, multifocality, rupture of the tumor at diagnosis).

However, for high-risk patients the treatment is difficult, and the disease-free survival rate was estimated near 29% in HB with small-cell undifferentiated histology, and about 55% in HB without small-cell undifferentiated histology (6).

Moreover, even though the chemotherapeutic treatment and the chemotherapy/surgery combined approach are the mainstay of treatment, incomplete response to chemotherapy and chemoresistance may induce unfavorable outcomes (7). For these reasons, there is still a need to investigate the cellular and molecular characteristics of HB, to better understand its mechanisms and to optimize stratification and therapies for patients with high risk (8).

A promising strategy to enrich our knowledge about HB biology and to discover new pathways for therapeutic tailoring could be based on the analysis of mechanistic similarities observed between this pediatric tumor and hepatocellular carcinoma (HCC), the most frequent primary liver cancer in adulthood. This approach has allowed the identification of different molecular pathways that could be useful for clinical decision-making for HB (9, 10). Among these pathways, the Wnt/β-Catenin (β-Cat) signaling is a tightly controlled molecular mechanism that regulates embryonic development, cellular proliferation, and differentiation, and it has been suggested that it could promote the development and/or progression of liver cancers, such as HCC and HB (11). The role of the β-Cat is amply discussed in HB, being found often mutated, and being its nuclear staining associated with an embryonal phenotype, thus predicting shorter survival rates (12, 13).

We recently reported that β-Cat gene expression positively correlated with the expression of the gene encoding for the focal adhesion kinase (FAK) in pediatric HCCs (14). FAK is a non-receptor tyrosine kinase encoded by the *PTK2* gene that, after its activation through auto-phosphorylation in the Tyr397 residue, contributes to the regulation of different cellular processes. FAK owes its name to its localization at focal adhesions, where upon integrin signaling is activated and takes part in the control of cell motility and directional cell migration. However, in the last two decades, it has been demonstrated that FAK can enter the nucleus via its nuclear localization signal to regulate different nuclear protein networks, probably acting as an adaptor protein or with other still unclear roles (15).

Several lines of evidence suggest a role of FAK in promoting different types of tumors, including HCC, where it has been reported that 26.1% of HCC tumors harbor FAK gene amplifications (16, 17). Moreover, FAK activity/signaling in adult and pediatric HCCs was associated with the control of epigenetic modulators (14). In particular, our previous data revealed a functional and/or physical interaction of FAK with enzymes involved in the control of histone H3 methylation and acetylation in HepG2 cells (18, 19).

Interestingly, Gillory et al. reported FAK protein expression and phosphorylation in HB samples (20), and characterization of genomic alterations in HB demonstrated gains in regions of chromosome 8 including also the locus encoding for *PTK2* (21, 22). These findings suggest that the correlation between FAK and epigenetic regulators deserves further investigation, especially in HB, where it could lead to the discovery of novel networks useful for targeted therapy.

In the present study, we analyzed the expression of FAK, β-Cat, and their epigenetic interactors in HB liver biopsies. Moreover, we investigated the effect of TAE226, a potent FAK inhibitor blocking the activation loop of the protein into a helical conformation (23), on Huh6 HB cells, to assess if this tumor cell type could benefit from its anti-tumorigenic effects.

## Materials and methods

### Patients’ samples

By reviewing the Pathology medical record, liver biopsies were obtained from a retrospective cohort of 17 patients with diagnosis of HB who underwent a surgical procedure to remove a portion of the liver at the “Bambino Gesù” Children’s Hospital (Rome, Italy) from 2011 to 2021. Two noncancerous liver samples were obtained from male donors (2 and 15 years old) at the time of transplantation. The study was approved by the institutional review board (protocol numbers OPBG_768.12, and OPBG_2070) which was in accordance with the Declaration of Helsinki, and written informed consent for biologic studies was obtained at the time of diagnosis from all patients analyzed.

### Histology and immunohistochemistry

Histological evaluation was performed on formalin-fixed and paraffin-embedded tissue 3 µm-thick sections and reviewed by two pathologists (PF and RA). Staining included hematoxylin and eosin, period acid-Schiff, period acid-Schiff after diastase digestion, and Masson trichrome using routine methods in liver pathology.

Immunohistochemistry was performed as already described (14), by using the following antibodies: monoclonal anti-β-catenin (Leica Biosystems, Germany), monoclonal anti-Glypican3-GPC3 (BioMosaics Inc., Burlington, VT, USA), and monoclonal anti-Glutamine Synthetase-GS (Millipore Inc., Billerica, MA, USA). The immunoreactivity for β-Cat (nuclear staining), GPC3, and GS (cytoplasmic staining) was semiquantitatively analyzed for the percentage of positive cells by a visual score calculated in at least 10 representative fields). For nuclear β-Cat scores were 0 (negative); 1 (positive); 2 (focal). For GPC3 and GS scores were: 0 (<5%); 1 (16–30%); 2 (31–60%); and 3 (>60%).

### RT-qPCR in liver tissue samples

Total RNA extraction from liver specimens was performed using RNeasy FFPE Kit (Catalog number 73504, Qiagen, Hilden, Germany) according to the manufacturer’s protocol. cDNA reverse transcription was conducted using the SuperScript VILO cDNA Synthesis kit (Catalog number 11754–050, Invitrogen-Thermo Fisher Scientific, Waltham, MA, USA). RT-qPCR amplification, detection and analysis were performed by QuantStudio 7 Pro RT-PCR System (Applied Biosystems-Thermo Fisher Scientific) using TaqMan Universal PCR Master Mix, No AmpErase UNG (Catalog number 4324018, Applied Biosystems-Thermo Fisher Scientific). The mRNA level expression of target genes was determined by using specific TaqMan commercial probes by Applied Biosystems-Thermo Fisher Scientific: these included FAK gene (*PTK2* Hs01056457_m1), β-Cat gene (*CTNNB1* Hs00355045_m1), AFP gene (AFP Hs01040598_m1), OCT4 gene (POU5F1 Hs04260367_gH), Epithelial cell adhesion molecule (EPCAM) gene (*EPCAM* Hs00901885_m1), and *SOX2* (SOX2 Hs01053049_s1). The mRNA levels were normalized to endogenous control gene eukaryotic 18S rRNA (18S Hs99999901_s1). The gene expression levels were represented as fold changes versus control and calculated by the ΔΔCt method.

### RNA sequencing and microarray datasets

Expression data were retrieved from Gene Expression Omnibus (GEO) database of National Center for Biotechnology Information (NCBI) (http://www.ncbi.nlm.nih.gov/geo/). For the analysis, five different datasets were used: GSE81928, GSE133039, GSE151347, and GSE104766 reporting high throughput sequencing gene expression data; and GSE131329 reporting microarray gene expression data. The analysis was conducted on the transcripts per million transcripts (TPM) normalized expression values for the GSE81928, GSE133039, GSE151347, and GSE104766 datasets; and on microarray expression values for the GSE131329 dataset.

### Immunofluorescence liver tissue samples

The 2µm slices were obtained from formalin-fixed paraffin-embedded liver tissue specimens. After dewaxing and rehydrating, heat-induced epitope retrieval was performed by boiling the slides with Dako Target Retrieval Solution EDTA (pH 9) (Dako-Agilent Technologies, Santa Clara, CA, USA). The primary antibodies were added and incubated overnight at 4°C (**Supplementary Material**, **Supplementary Table S1** for the list of antibodies used). The primary antibodies were revealed with the secondary antibodies Alexa Fluor 488 (**Supplementary Material**, **Supplementary Table S1**). Cell nuclei were counterstained with DAPI. Samples were digitalized by using the Hamamatsu Nanozoomer S60 Digital slide scanner C13210–01 (Hamamatsu Photonics, Shizuoka, Japan), equipped with an Olympus 20X/0.75 and 40X/1.40 PlanSApo objectives (Olympus, Tokyo, Japan), a Fluorescence Imaging Module with a LX2000 mercury lamp (Hamamatsu Photonics), a linear ORCA-Flash 4.0 digital CMOS camera (Hamamatsu Photonics). Whole slide images (WSIs) were used to manually draw the region of interest (ROI) and perform quantitative fluorescence imaging analysis (QFIA). The intensity average of fluorescence was calculated using ImageJ software, version 1.8.0 (National Institutes of Health, Bethesda, MD, USA). Images were also acquired by confocal microscopy performed on an Olympus Fluoview FV3000 Confocal Laser Scanning Microscope (Olympus).

### Experimental methods in cells

Detailed materials and methods for all the experimental procedures used for the cellular model are available in **Supplementary Materials and Methods** (Cells and treatment, Real-Time Monitoring of Cell Proliferation, Cell Viability Assay, Clonogenic Assay, Confocal cellular microscopy, Annexin V assay by flow cytometry (FACS), Real-Time Monitoring of Cell Apoptosis, Cell extracts, Western Blotting, RT-qPCR in HB cells).

### Statistics

All data are presented as mean ± standard deviation. Comparisons between two groups were performed by paired or unpaired two-tailed Student’s t-test or t-test with Welch’s correction. Pearson and Spearman correlation analyses were performed to test the association between gene and protein expression and clinical features on two-way contingency tables. p < 0.05 was considered to be significant. Statistical analyses were undertaken using GraphPad Prism, version 9 for Windows (GraphPad Software Inc., San Diego, CA, USA).

## Results

### FAK gene is over-expressed in liver tissues from human HB

**Figure 1:**
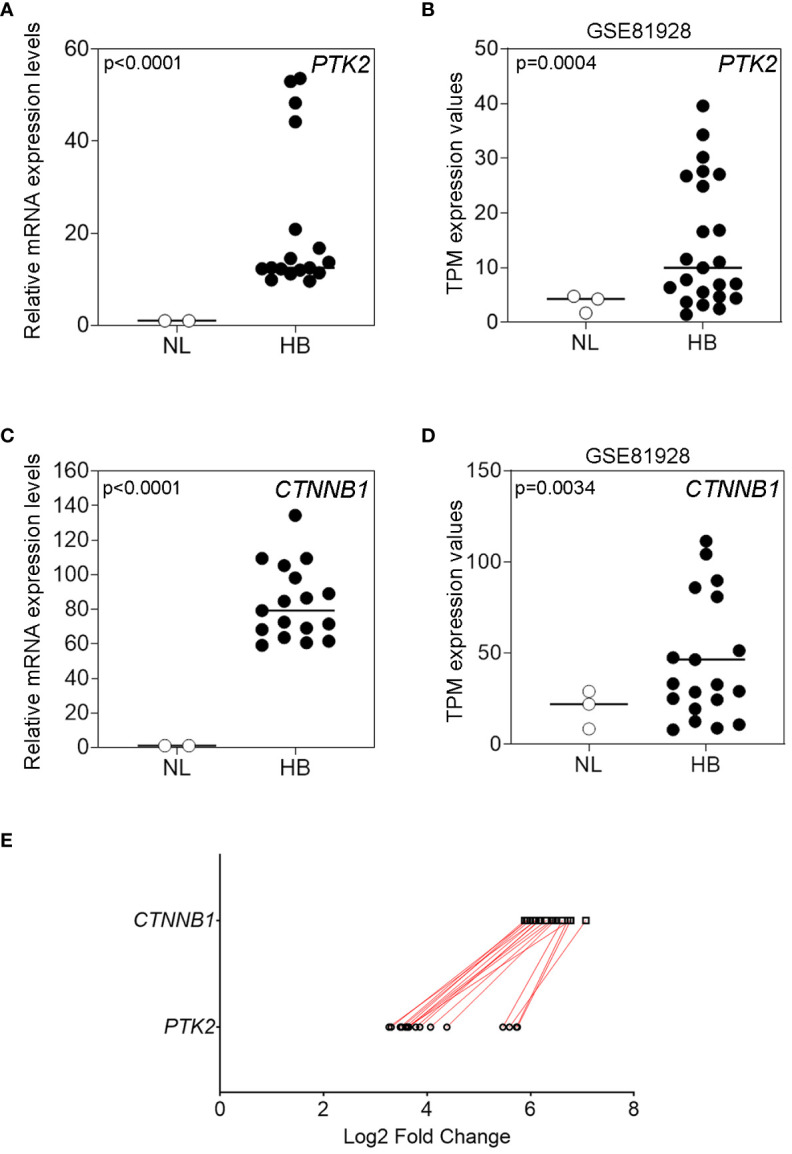
FAK and β-Cat gene expression in HB tissues and in normal liver (NL). **(A)** Relative mRNA expression of *PTK2* measured by RT-qPCR in NL (n=2) and in pediatric HB (n=17) tissues; **(B)** TPM expression values of *PTK2* in the GSE81928 RNA-sequencing dataset in NL (n=3) and in HB (n=23) tissues. **(C)** Relative mRNA expression of *CTNNB1* measured by RT-qPCR in NL (n=2) and in pediatric HB (n=17) tissues; **(D)** TPM expression values of *CTNNB1* in the GSE81928 RNA-sequencing dataset in NL (n=3) and in HB (n=23) tissues. Bars indicate the median. **(E)** Comparison of the Log2 fold changes assessed by RT-qPCR of *PTK2* and *CTNNB1* relative expression levels in pediatric HB (n=17) tissues. The red lines connect data from the same patient.

In a similar way to what previously investigated in pediatric HCC (14), here we investigated the FAK gene/protein expression in children affected by HB. The study cohort included 17 patients with HB diagnosis (9 males and 8 females) with a median age of 19 months (ranging from 2 to 127 months). We firstly performed the analysis of FAK gene (*PTK2*) expression in 17 HB compared to normal liver (NL) tissues from 2 healthy donors by RT-qPCR. *PTK2* was significantly (p<0.0001) over-expressed in HB tissues compared to NL tissues (**Figure 1A**). In line with our results, *PTK2* was found to be significantly (p=0.0004) up-regulated also in the GSE81928 RNA-sequencing dataset that compared 23 HB and 3 NL (**Figure 1B**). Also, *CTNNB1* expression was higher in HB than in NL in both our cohort (p<0.0001) (**Figure 1C**) and in GSE81928 dataset (p=0.0034) (**Figure 1D**). Similarly, the analysis performed in other GEO datasets highlighted that *PTK2* expression and *CTNNB1* were significantly up-regulated in tumor samples from HB compared to the adjacent non-cancerous liver (NCL) tissues (**Supplementary Material**, **Supplementary Figures S1A–H**).

Furthermore, the comparison of *PTK2* with *CTNNB1* expression for each sample showed a concomitant up-regulation of these genes (**Figure 1E**).

### FAK functional activation correlated with alterations of epigenetic regulators, AFP levels and tumor size in human HB

**Figure 2:**
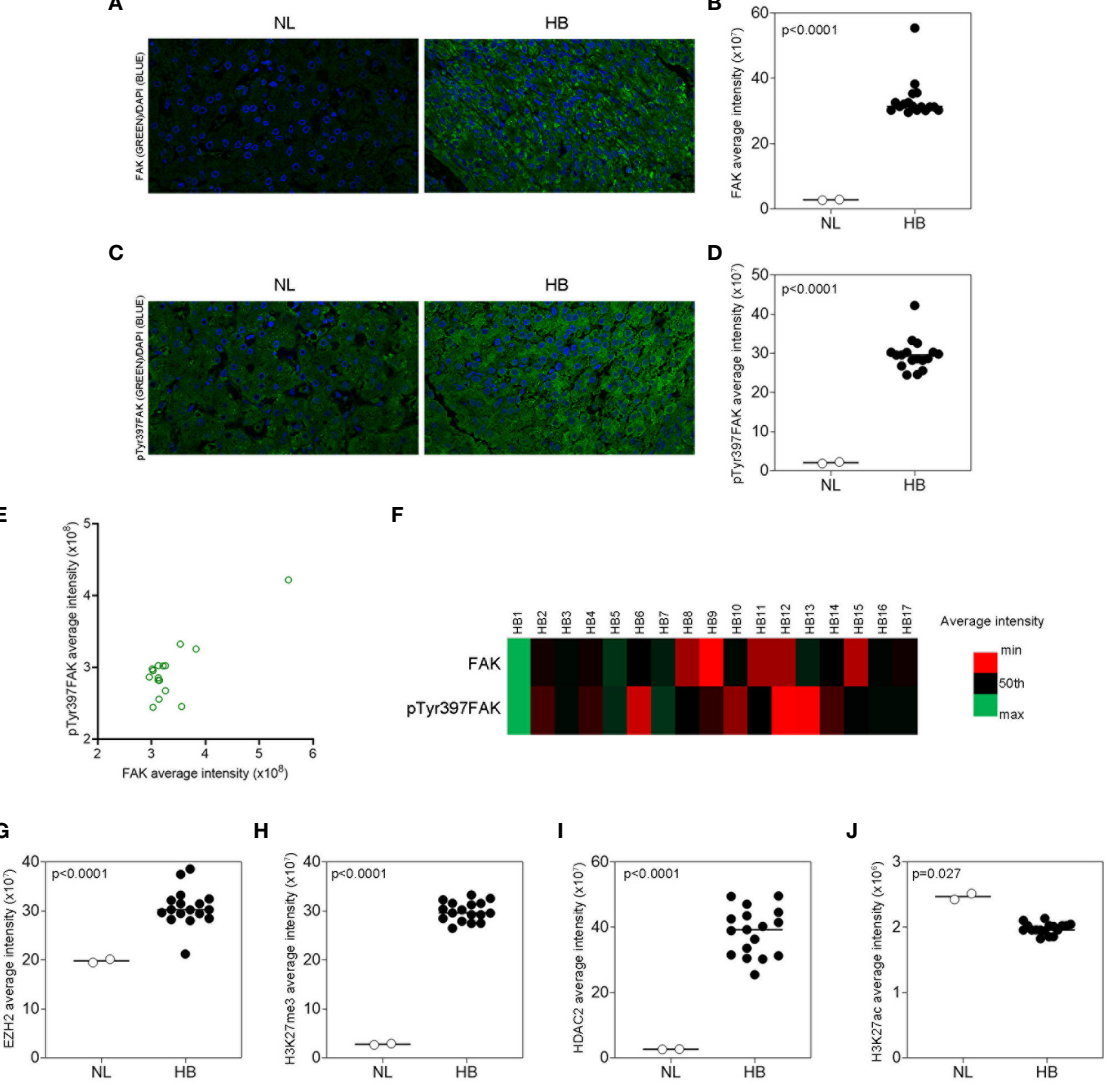
FAK and epigenetic regulators protein expression in HB and in NL tissues. **(A, B)** Representative immunofluorescence and average fluorescence intensity calculated for FAK (green) in NL and in HB tissues. **(C, D)** Representative immunofluorescence and average fluorescence intensity calculated for pTyr397FAK (green) in NL and in HB tissues. DAPI (blue) for nuclear staining. 40X magnification. Scatter plot **(E)** and heatmap **(F)** representations of the correlation between total FAK and pTyr397FAK protein expression in pediatric HB (n=17) tissues. Average fluorescence intensity calculated for EZH2 **(G)**, H3K27me3 **(H)**, HDAC2 **(I)**, and H3K27ac **(J)** in NL and HB tissues. Bars in the histograms indicate the medians.

Next, FAK protein expression was evaluated by WSI and QFIA. As shown in **Figures 2A, B**, FAK protein was significantly over-expressed (p<0.0001) in HB with respect to NL tissues. Furthermore, we evaluated the expression of FAK activated form (pTyr397FAK) in our HB and NL tissues. QFIA analysis revealed in HB a higher expression of pTyr397FAK compared to NL samples (**Figures 2C, D**). As shown in **Figures 2E, F**, pTyr397FAK expression positively correlated with total FAK expression (r=0.81, p<0.0001) in HB samples. Beside the well-established role of FAK in modulating cell adhesion, migration, and proliferation in HCC, our recent studies have shown its networking with epigenetic mechanisms, including histone H3 methylation and acetylation (18, 19). Therefore, we explored the possible association of the expression of FAK and pTyr397FAK with the regulation of histone H3 lysine 27 trimethylation (H3K27me3) and acetylation (H3K27ac) by two crucial epigenetic regulators, the N-methyltransferase EZH2 and the histone deacetylase 2 (HDAC2) that removes acetyl groups from H3 histone (24). In particular, we also evaluated the protein expression of EZH2, H3K27me3, HDAC2, and H3K27ac by WSI and QFIA in our HB and NL samples. As shown in **Figures 2G–I**, the expression of EZH2, H3K27me3, and HDAC2 was significantly up-regulated (p<0.0001) in HB tissues with respect to NL samples. On the other hand, the expression of H3K27ac was significantly (p=0.027) down-regulated in HBs in comparison to NLs (**Figure 2J**).

Finally, we evaluated the correlation of FAK and pTyr397FAK expression with epigenetic regulators and HB clinical features, including nuclear expression of β-Cat, AFP levels, presence of metastasis, tumor size, histology subtype, and outcome. Anthropological and clinical features of patients are reported in **Supplementary Material**, **Supplementary Table S2**. Thirteen HB exhibited a nuclear β-Cat protein stained by immunohistochemistry (**Supplementary Material**, **Supplementary Figure S2A**). According to the AFP threshold suggested by a recent meta-analysis (25), high AFP levels (≥400 ng/mL) were detected in all cases. Sixteen patients exhibited a single nodule with a size that was <5 cm in 7 patients, and ≥5 cm in 10 patients. Three cases had fetal histological phenotype, 3 cases had embryonal histological phenotype, 9 cases were mixed fetal and embryonal, whereas 2 cases were diagnosed as hepatocellular neoplasm-NOS.

Correlation analysis showed no correlation between nuclear expression of β-Cat protein and FAK or pTyr397FAK expression (**Supplementary Material**, **Supplementary Figure S2B, C**), thus β-Cat was excluded from the next correlation analyses. As reported in **Table 1**, the Spearman correlation analysis showed that only the pTyr397FAK expression was significantly associated with AFP levels, tumor size, and with the expression of epigenetic regulators EZH2 and H3K27me3. Moreover, EZH2 and H3K27me3 expression correlated each other and both with AFP levels and tumor size. Finally, HDAC2 expression was significantly positively correlated with AFP levels, even though its negative correlation trend with H3K27ac didn’t reach the significance (r=-0.321; p=0.208).

**Table 1:** Table showing Spearman's rho correlation and p values (round brackets) for all parameters.

	AFP levels	Tumor size	Metastasis	Histotype	FAK	pTyr397FAK	EZH2	H3K27me3	HDAC2
***Tumor size***	0.461 (0.063)								
***Metastasis***	0.254 (0.350)	0.340 (0.187)							
***Histotype***	-0.0133 (0.961)	0.333 (0.190)	-0.046 (0.998)						
***FAK***	0.002 (0.994)	0.183 (0.477)	0.255 (0.334)	-0.229 (0.375)					
***pTyr397FAK***	**0.579 (0.016)**	**0.679 (0.003)**	0.141 (0.602)	0.066 (0.797)	0.325 (0.200)				
***EZH2***	**0.612 (0.010)**	**0.542 (0.026)**	0.170 (0.526)	0.115 (0.656)	0.3703 (0.143)	**0.782 (0.0003)**			
***H3K27me3***	**0.680 (0.003)**	**0.641 (0.006)**	0.382 (0.136)	-0.019 (0.941)	0.387 (0.124)	**0.679 (0.003)**	**0.713 (0.001)**		
***HDAC2***	**0.556 (0.022)**	0.308 (0.226)	-0.028 (0.956)	-0.169 (0.514)	-0.018 (0.945)	0.448 (0.072)	0.437 (0.080)	0.457 (0.066)	
***H3K27Ac***	-0.004 (0.988)	-0.324 (0.202)	-0.084 (0.784)	-0.025 (0.924)	-0.414 (0.098)	-0.218 (0.396)	-0.143 (0.579)	-0.268 (0.294)	-0.321 (0.208)

Significant rho and p values are reported in bold.

### Effect of FAK inhibitor TAE226 on cell growth in Huh6 human HB cells

**Figure 3:**
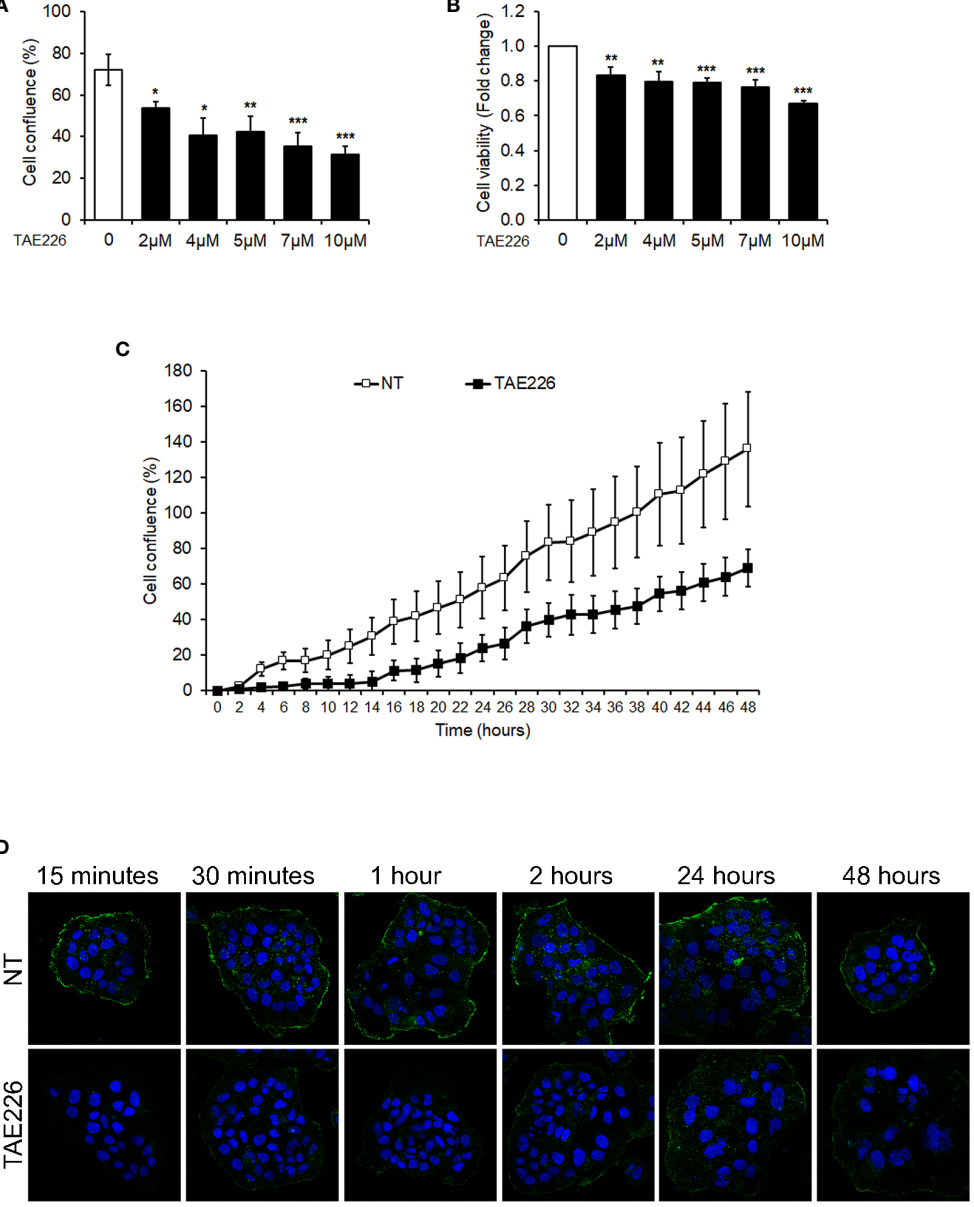
The FAK inhibitor TAE226 reduces cellular growth and pTyr397FAK protein levels of Huh6 human HB cells. **(A)** Cell proliferation monitored by using the Incucyte Live Cell Imaging system, expressed as percentage of cell confluence, in Huh6 cells untreated or treated with TAE226 at different concentrations for 48 hours; **(B)** cell viability expressed as fold change of absorbance values by XTT assay, in Huh6 cells untreated or treated with TAE226 at different concentrations for 48 hours; **(C)** growth curves analyzed as percentage of cell confluence through Incucyte Live Cell Imaging system and monitored every 2 hours for 48 hours in Huh6 cells non-treated (NT) or treated with 7µM TAE226 (TAE226); **(D)** representative immunofluorescence by confocal imaging of pTyr397FAK (green) in Huh6 cells NT or TAE226 at different timepoints. 60X magnification. Hoechst nuclear staining (blue). Data were normalized respect to the number of cells at the time 0. Values are the mean ± SD of three independent experiments repeated at least in triplicate. Data were analyzed by 2-tailed Student’s t test. *p < 0.05; **p < 0.01; ***p < 0.001.

In a previous study, we demonstrated that the FAK inhibitor TAE226 was the most effective in reducing HCC growth both alone and in combination with sorafenib, enhancing its antitumor activity and overcoming mechanisms of resistance (19). Therefore, we sought to investigate the effects of FAK inhibition through this molecular compound also in the Huh6 human HB cell line. We first analyzed the effect of TAE226 on cell proliferation and viability. We tested different concentrations (2, 4, 5, 7, and 10µM) of TAE226 to treat Huh6 cells. The analysis of cell proliferation through live imaging revealed that after 48 hours of treatment, the inhibitor had a dose-dependent significant anti-proliferative effect (**Figure 3A**), without inducing evident cytotoxicity tested by XTT assay (**Figure 3B**).

In particular, the real-time monitoring of cell growth from 0 to 48 hours highlighted that the cell confluence was halved in 7µM TAE226-treated Huh6 cells with respect to non-treated (NT) counterpart (**Figure 3C**; **Supplementary Figure S3A**). The anti-proliferative effect of 7µM TAE226 was confirmed through a clonogenic assay at different timepoints (**Supplementary Figure S3B**). Furthermore, 7µM TAE226 caused at 15 minutes an immediate reduction of the expression of pTyr397FAK, and this effect was maintained until 48 hours from the treatment (**Figure 3D**; **Supplementary Figure S3C**). Therefore, the 7µM TAE226 (TAE226) concentration was selected as the dose to administer to Huh6 cells for the next experiments.

### Effects of FAK inhibitor TAE226 on apoptosis in Huh6 human HB cells

**Figure 4:**
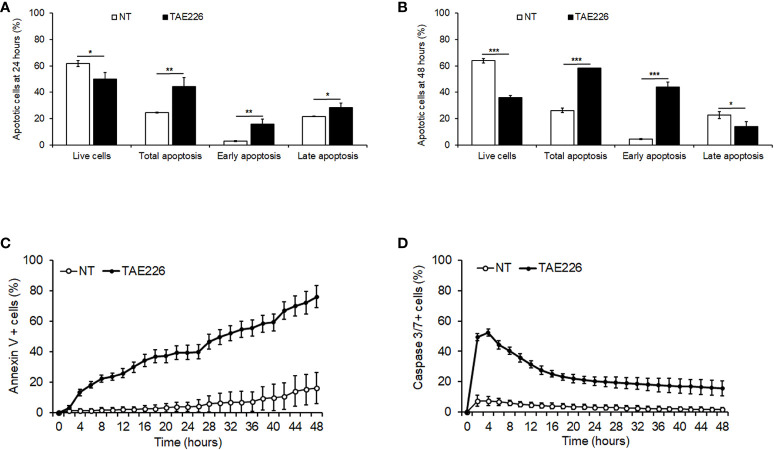
The FAK inhibitor TAE226 induces apoptosis in Huh6 human HB cells. Percentage of live or apoptotic cells measured by Annexin V and PI staining by FACS in Huh6 cells NT or treated with TAE226 for 24 hours **(A)** and 48 hours **(B)**. Percentage of Annexin V **(C)** and Caspase 3/7 **(D)** positive cells monitored every 2 hours for 48 hours by Incucyte Live Cell Imaging system and analyzed for quantification of green fluorescent objects in Huh6 cells NT or treated with TAE226. Data were normalized respect to the number of positive cells at initial time (0 hour). Values are the mean ± SD of three independent experiments and were analyzed by 2-tailed Student’s t test. *p < 0.05; **p < 0.01; ***p < 0.001 vs NT.

We further sought to determine if the FAK inhibitor TAE226 effect on the growth of Huh6 human HB cells was also associated with the induction of apoptosis. The analysis by FACS with Annexin V/PI staining revealed that after 24 and 48 hours 7µM TAE226 was able to increase significantly the percentage of total apoptotic cells with respect to NT cells, mainly by causing a significant induction of early apoptosis (**Figures 4A, B**; **Supplementary Figures S4A, B**). This pro-apoptotic effect of TAE226 was also confirmed through the real-time system for monitoring Annexin V and Caspase 3/7 expression in living cells every 2 hours in a timeframe ranging from 0 to 48 hours (**Figures 4C, D**; **Supplementary Figures S4C, D**).

### Effects of FAK inhibitor TAE226 on epigenetic regulators and cancer-related stemness genes in Huh6 human HB cells

**Figure 5:**
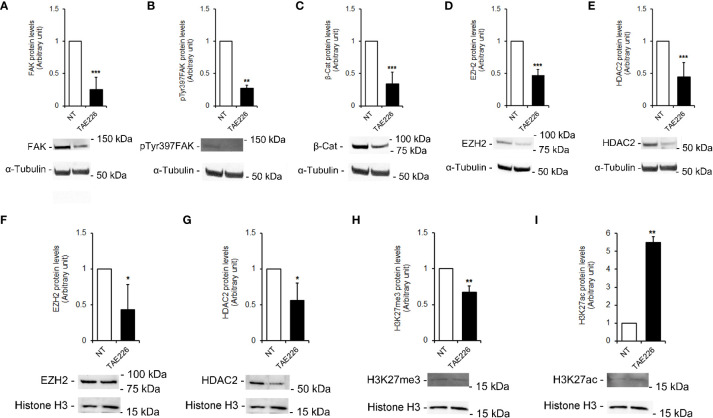
FAK inhibitor TAE226 effects on protein levels of epigenetic regulators in Huh6 human HB cells. Representative immunoblot and quantitative analysis of FAK **(A)**, pTyr397FAK **(B)**, β-Cat **(C)**, EZH2 **(D)**, HDAC2 **(E)** protein levels in whole cell extracts from Huh6 cells NT or treated with TAE226; representative immunoblot and quantitative analysis of EZH2 **(F)**, HDAC2 **(G)**, H3K27me3 **(H)**, and H3K27ac **(I)** protein levels in nuclear extracts from Huh6 cells NT or treated with TAE226. α-Tubulin and histone H3 served as loading controls for whole lysate and nuclei, respectively. Values are the mean arbitrary units ± SD of at least three independent experiments. Data were analyzed by 2-tailed Student’s t test. *p < 0.05; **p < 0.01; ***p < 0.001 vs. NT.

Our previous studies in HCC demonstrated the effect of FAK inhibition by silencing or TAE226 treatment in affecting the expression of tumor-promoting genes through deregulation of multiple epigenetic regulators, such as EZH2 and HDAC2 (18, 19). As reported above, those molecules have been found altered also in HB tissues (see **Figure 2**). Indeed, here we investigated whether the TAE226 treatment was effective in improving the levels of these epigenetic regulators in Huh6 cells. In particular, we performed Western blotting in whole cell lysates and in nuclear extracts from NT or TAE226-treated Huh6 cells after 48 hours from treatment. Our results revealed a significant effect of TAE226 in decreasing the whole cells protein levels of total FAK (**Figure 5A**) and pTyr397FAK (**Figure 5B**). In addition, under TAE226 administration we observed reduced whole cell levels of β-Cat (**Figure 5C**), EZH2 (**Figure 5D**), HDAC2 (**Figure 5E**) proteins; decreased nuclear protein levels of EZH2 (**Figure 5F**), HDAC2 (**Figure 5G**), H3K27me3 (**Figure 5H**); and increased nuclear protein levels of H3K27ac (**Figure 5I**).

**Figure 6:**
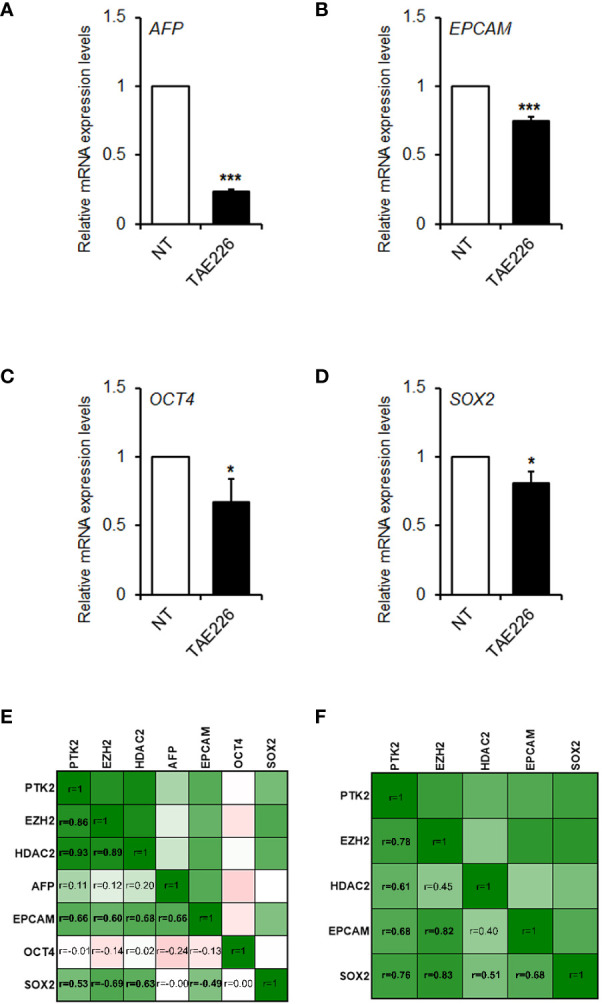
FAK is correlated with the expression of stemness genes in HB. Relative mRNA expression of *AFP* **(A)**, *EPCAM* **(B)**, *OCT4* **(C)**, and *SOX2* **(D)** genes measured by RT-qPCR in Huh6 cells NT or treated with TAE226. Values are the mean arbitrary units of the relative mRNA expression ± SD of at least three independent experiments. Data were analyzed by 2-tailed Student’s t test. *p < 0.05; ***p < 0.001 vs. NT. **(E)** Heatmap showing the Pearson’s correlation matrix among the gene expression levels of *PTK2*, *EZH2*, *HDAC2*, *AFP*, *EPCAM*, *OCT4* and *SOX2* in GSE81928 HB tissues. **(F)** Heatmap showing the Pearson’s correlation matrix among the gene expression levels of *PTK2*, *EZH2*, *HDAC2*, *EPCAM*, and *SOX2* in our set of HB tissues. The values of correlation coefficients (r) were reported, and significant correlations were highlighted in bold. All p values were <0.05.

Finally, stemness markers are strongly expressed in HB, signifying it as a kind of tumor with remarkable stemness properties. Previous lines of evidence highlighted that FAK may modify the expression of key genes involved in reprogramming and cancer stemness acquisition in liver cancer cells (26, 27), including HepG2, a cell line of hepatocellular neoplasm, which retain HB features (28). Therefore, here we evaluated the effect of TAE226 on a group of crucial liver cancer-related stemness genes in Huh6 human HB cells. As showed in **Figures 6A–D**, TAE226 resulted effective in down-regulating the expression of *AFP*, *EPCAM*, *OCT4*, and *SOX2* genes, in Huh6 cells treated with TAE226 respect to NT cells.

Finally, we analyzed the correlation between gene expression levels of FAK, its epigenetic interactors and stemness genes, both in our cohort of patients and in GSE81928 dataset. As shown in **Figure 6E**, *PTK2* expression significantly correlated with *EZH2*, *HDAC2*, *EPCAM*, and *SOX2* in GSE81928 samples. The consistency of these correlations was also evaluated in our HB tissues, where we obtained similar results (**Figure 6F**).

## Discussion

In this study, we evaluated the expression and activity of FAK and its related epigenetic network in HB samples and one HB cell line. We found a significant up-regulation of FAK mRNA and protein expression in livers from HB compared to normal livers. In HB tissues, FAK mRNA expression levels correlated with β-Cat mRNA expression levels. Whereas, the increased protein expression of total FAK was correlated with the increase of its Tyr397 phosphorylated/active form, which was significantly correlated with the expression of some epigenetic regulators of histone H3 methylation and acetylation, and with AFP levels and tumor size. Moreover, *in vitro* results demonstrated that FAK inhibitor TAE226 caused a reduction of Tyr397 phosphorylated FAK, β-Cat and epigenetic regulators protein levels, in association with evident anti-proliferative and pro-apoptotic effects on HB cells, and with down-regulation of a group of liver cancer-related genes with a key role in stemness.

According to previous studies in HCC that describe a FAK/β-Cat axis (14, 29–31), here we found a similar trend in HB. Indeed, the expression of the two forms of FAK was extensively investigated in HCC, where they correlated with tumor phenotype and β-Cat localization (30). Moreover, the same authors reported an association between FAK over-expression and β-Cat mutations in the 34.8% of human HCC tissues (31). In our previous study, we demonstrated a significant correlation between FAK and β-Cat over-expression in pediatric HCC tissues, in particular in the presence of a cirrhotic background (14). Previous studies suggest that *PTK2* gene up-regulation and consequent protein over-expression could depend on a frequent copy number alteration of chromosome 8/8q found in HB tissues (22), but also by other indirect mechanisms that could involve the regulation via non-coding RNAs, such as IGF2/H19 axis (32). However, these mechanisms have not been explored in our samples.

Only a previous study by Gillory et al. (20) reported the expression of total and Tyr397 phosphorylated FAK in HB tumor specimens. Here, we demonstrated for the first time that the expression of these forms of FAK protein was higher in HB samples than in normal livers. However, our data in HB showed that the expression of FAK and Tyr397FAK was not correlated with β-Cat nuclear expression, tumor phenotype (i.e. embryonal, fetal or epithelial-mesenchymal), or metastasis (1). On the contrary, the expression of Tyr397FAK positively correlated with tumor size and AFP levels, thus suggesting that an analysis of FAK active form in HB requires further investigations on a larger sample size in line with PRETreatment EXTent (PRETEXT) disease classification that considers also tumor extension (33).

According to epigenetic landscape evaluated in HB by Clavería-Cabello et al. (34), in our HB samples we observed an altered expression of two histone-modifying enzymes. In particular, we observed an up-regulation of EZH2 concomitant to an up-regulation of H3K27me3. In addition, it was found an up-regulation of HDAC2 apparently uncoupled to a significant down-regulation of H3K27ac. This latter discrepancy could be due to the small sample size, but we also speculate that this may depend on the fact that H3K27ac is under the control of multiple HDACs. Anyway, further evaluation of RNA-Seq and ChiP-Seq signatures could be crucial to establish if changes of histone-modifying enzymes effectively alter epigenomic phenotype in HB context.

Interestingly, we recently identified a possible nexus between these epigenetic regulators and nuclear FAK by direct or indirect interactions (19). Of note, here, we found that in HB tissues the expression of FAK and pTyr397FAK was correlated with the expression of the two epigenetic regulators EZH2 and HDAC2, and their downstream effectors. In addition, more importantly, pTyr397FAK, EZH2, and H3K27me positively correlated with tumor size and AFP levels, thus suggesting that FAK and its epigenetic network in HB deserve further studies in more HB samples and models.

Several lines of evidence suggest that FAK is a key actor not only in controlling cell adhesion, migration, proliferation, survival, and invasion (17) but also as a regulator of gene expression (35). In particular, understanding the crosstalk between FAK and epigenetic modifications in cancer is a rapidly evolving field. Indeed, FAK scaffolding functions can influence microRNAs expression, chromatin remodeling, and post-translational histone modifications (36). In our previous studies, we found an involvement of FAK in epigenetic modifications of HCC, and we showed that its depletion, through pharmacological inhibition or silencing reduced HCC growth *in vivo* and *in vitro* in different liver cancer cell lines (18, 19). In particular, among the inhibitors we found as the most efficient the TAE226, which is a dual inhibitor of FAK and insulin-like growth factor 1 receptor (IGF-1R) that demonstrated to have promising anti-tumorigenic effects in several malignancies (37). Here we showed that TAE226 FAK inhibitor was effective in decreasing cell growth, and in increasing apoptosis also in an undoubted Huh6 HB cell line. This corroborates previous studies on the crucial role of FAK as an oncogene also in HB and the potential effects of FAK inhibitors (18, 19, 38, 39). Here, we reported that TAE226 reduces the expression of EZH2 and HDAC2 with a consequent decrease of H3K27me3 and an increase of H3K27ac. In line with this evidence, in our previous studies we demonstrated a FAK intertwining with EZH2 (18) and/or some components of the Nucleosome Remodelling and Deacetylase (NuRD) complex, a chromatin remodeling complex with histone deacetylase activity (19). A definition of the interconnection between FAK and epigenetics deserves further studies in animal models. Indeed, it could be key to better explore these interactions also in HB, where several studies suggested that epigenetic factors, such as methylation profile, could be useful molecular biomarkers for HB risk stratification, and its systematic molecular characterization may be crucial to understand the epigenetic driver events that occur in HB development (9, 40, 41).

Our findings, even if preliminary, suggest that TAE226 may cause a deregulation of FAK and its epigenetic networks also in HB. In turn, FAK-related changes in epigenetic regulation may act on target genes, such as those associated with cancer stemness properties (26, 27). Indeed, here we found that inhibition of FAK phosphorylation/activity in HB cells was able to reduce the expression of *AFP*, *EPCAM*, *OCT4*, and *SOX2*, considered as crucial liver cancer stem cell genes (42). Accordingly, in GSE81928 dataset and in our HB samples the expression of *PTK2* gene correlated with the expression of *AFP* and *SOX2*. Overall, as suggested by other authors, our findings confirm that some HBs could exhibit similar HCC features with an evident association with FAK and that should be deeply investigated in order to understand their relevance for clinical outcome and therapy (13, 43).

Overall, our findings remain exploratory highlighting some interesting molecular aspects, thus a more in deep mechanistic elucidation in *in vivo* and *in vitro* models is required.

In conclusion, our results suggest that FAK overexpression/activation could be a relevant marker in HB even though the lacking of comparisons between tumor area and adjacent non-tumor area in a same patient remains a limit of our study. Moreover, whether FAK evaluation can be implemented in the clinical routine as diagnostic or prognostic marker in HB remains to be seen in studies with a larger sample size. Finally, our results sustain also the need of a mechanistic dissection of the dysregulation of all epigenetic processes linked to FAK to better define the effective therapeutic potentiality of FAK inhibitors alone or in combination with epigenetic therapies.

## Data availability statement

The original contributions presented in the study are included in the article/**Supplementary Material**. Further inquiries can be directed to the corresponding author.

## Ethics statement

The studies involving humans were approved by Bambino Gesù Children’s Hospital. The studies were conducted in accordance with the local legislation and institutional requirements. Written informed consent for participation in this study was provided by the participants’ legal guardians/next of kin.

## Author contributions

MRB: Investigation, Formal analysis, Writing – review & editing, Writing – original draft, Methodology, Data curation, Conceptualization. CDS: Software, Methodology, Formal analysis, Writing – review & editing, Investigation, Data curation. FT: Investigation, Writing – review & editing, Formal analysis, Data curation. AC: Writing – original draft, Data curation. NC: Investigation, Writing – original draft, Data curation. MP: Methodology, Writing – original draft, Data curation. VT: Writing – original draft, Methodology, Data curation. MS: Writing – review & editing, Data curation. RA: Writing – review & editing, Writing – original draft, Methodology. AA: Software, Writing – review & editing, Writing – original draft, Supervision, Resources, Project administration, Investigation, Funding acquisition, Data curation, Conceptualization. PF: Writing – review & editing, Writing – original draft, Validation, Methodology, Investigation, Data curation.
