# Supplementary material for: Focal adhesion kinase and its epigenetic interactors as diagnostic and therapeutic hints for pediatric hepatoblastoma
Source: Front Oncol. 2024 Jun 14;14:1397647. doi: 10.3389/fonc.2024.1397647 (PMC11211568; doi:10.3389/fonc.2024.1397647)
Supplement: Supplementary file 1 [file DataSheet_1.docx]

Supplementary Material

**Focal adhesion kinase and its epigenetic interactors as diagnostic and therapeutic hints for pediatric hepatoblastoma**

Maria Rita Braghini^1†^, Cristiano De Stefanis^2†^, Francesca Tiano^1^, Aurora Castellano^3^, Nicolo’ Cicolani^2^, Marco Pezzullo^2^, Valeria Tocco^2^, Marco Spada^4^, Rita Alaggio^5‡^, Anna Alisi^1*‡^ and Paola Francalanci^5‡^

***Correspondence:** Corresponding Author: Anna Alisi [anna.alisi@opbg.net](mailto:anna.alisi@opbg.net)

# Supplementary Material and Methods

**Cells and Treatment**

Human HB cells Huh6 were purchased from JCRB Cell Bank and maintained in DMEM supplemented with 10% FBS and 1% penicillin and streptomycin (Gibco-Thermo Fisher Scientific) at 37°C under 5% CO2 in a 95% humidified atmosphere. Cells were screened for possible mycoplasma contamination by using Venor GeM Advance Mycoplasma Detection KIT (Minerva Biolabs GmbH, Berlin, Germany). All the experiments were performed only in mycoplasma-free cells. TAE226 treatment was kindly provided by Novartis Pharma (Novartis Pharma, Basel, Swiss) and was dissolved in dimethyl sulfoxide and stored at -80°C until the time of use. Cells were treated with different concentrations of TAE226 (2, 4, 5, 7 and 10 µM) for 48 hours to evaluate the effect of the treatment on cell viability and proliferation and to establish the most effective dose treatment.

**Real-Time Monitoring of Cell Proliferation**

Real-time cell proliferation changes were measured by using an Incucyte label-free cell proliferation assay (Sartorius, Göttingen, Germany). Huh6 cells were seeded in quintuplicate at a density of 8,000 cells per well in 96-well plates in 200μL of complete medium and incubated at 37°C overnight. Subsequently, cells were treated or not with different concentrations of TAE226 and placed into the Incucyte live-cell analysis system (Sartorius). The time-lapse imaging has been set to acquire four images per well from five technical replicates every 2 hours by using a 10X objective lens over a time course of 48 hours, and then classic confluence analysis was performed by using IncuCyte basic software, version 2021A (Sartorius).

**Cell Viability Assay**

Cell viability was evaluated by using the cell proliferation kit II-XTT (Hoffmann-La Roche, Basel, Swiss) according to the manufacturer’s protocol. In particular, 10,000 cells per well were seeded in a 96-well culture plate in quintuple. At the end of the treatment, a mixture of XTT labeling reagent and electron-coupling reagent was added in the media, and cells were incubated at 37°C for 4 hours. The absorbance of the water-soluble formazan formed was measured at 492 and 620 nm using an ELISA microplate spectrophotometer. Three independent experiments were conducted.

**Clonogenic Assay**

Huh6 cells were seeded at a density of 2x106 cells into 100 mm cell culture dishes in a complete medium and then treated or not with 7μM TAE226 for 48 hours. Untreated and TAE226-treated cells were subsequently collected by using trypsin-EDTA and seeded at a density of 300,000 cells into 60 mm cell culture dishes (five replicated of each) in a complete medium. Petri dishes were then incubated for various incubation times (0, 5, 10, 15, 20 days). When colonies formed, the experiment was stopped by gently washing with PBS and fixing with 10% formalin solution for 10 minutes, and finally, cells were stained with 0.01% crystal violet solution (Catalog number C0775, Sigma Aldrich-Merck KGaA, Darmstadt, Germany) for 15 minutes, washing excess with ddH2O and allowing dishes to dry.

**Confocal cellular microscopy**

Huh6 cells were cultured in 4-well chamber slides and treated with TAE226 for different timepoints. Next, cells were fixed with 4% paraformaldehyde in PBS for 10 minutes. After two brief washes with PBS, cells were blocked with PBS/BSA 1% at room temperature (RT) for 30 min and then incubated with the primary antibody (supplementary material, **Table S1**) diluted in PBS/BSA 1% overnight at 4°C. Then, cells were washed twice with PBS and incubated with the secondary antibody Alexa Fluor 488 (supplementary material, **Table S1**) in PBS/BSA 1% for 1 hour at RT. Next, cells were washed with PBS and incubated with 1:10000 Hoechst in PBS for 10 minutes at RT for nuclear staining. Imaging was performed by using the original digital images format acquired with an Olympus Fluoview FV3000 Confocal Laser Scanning Microscope (Olympus).

**Annexin V assay by flow cytometry (FACS)**

Apoptosis was assessed by FITC Annexin V Apoptosis Detection Kit I (Catalog number 556547, Becton Dickinson-BD, Franklin Lakes, NJ, USA). Cells were seeded at a density of 300,000 cells/well in a 6-well plate and then treated or not with 7µM TAE226. Next, cells were washed twice with cold PBS and resuspended in 1X Annexin Binding Buffer. Cells were then stained with 5μL of FITC Annexin V and with 5μM Propidium Iodide (PI) for 15 minutes before analyzing. Acquisition and analysis were carried out on a FACS Canto II flow cytometer, using DiVa Software, version 6.3 (Becton Dickinson-BD).

**Real-Time Monitoring of Cell Apoptosis**

Cell apoptosis was evaluated by real-time monitoring the expression of Annexin V and Caspase 3/7 by using an Incucyte Apoptosis Assay with Incucyte Annexin V Green Reagent (Catalog number 4642, Sartorius) and Incucyte caspase-3/7 Green Apoptosis Reagent (Catalog number 4440, Sartorius), respectively. Huh6 cells were seeded in quintuplicate at a density of 8,000 cells per well in 96-multiwell plates in 200μL of complete medium and incubated at 37°C overnight. Subsequently, cells were treated or not with 7μM TAE226 and placed into the Incucyte live-cell analysis system (Sartorius). The time-lapse imaging has been set to acquire four images per well from five technical replicates every 3 hours by using a 10X objective lens over a time course of 48 hours, and then the analysis of green fluorescence was performed using the Incucyte Cell-by-Cell Analysis Software (Sartorius).

**Cell extracts**

For protein extraction, Huh6 cells were plated in 100 mm cell culture dishes in a complete medium and then treated or not with 7μM TAE226 for 48 hours. Whole-cell lysates were collected by using trypsin-EDTA and recovered in RIPA lysis buffer (Catalog number R0278, Sigma Aldrich-Merck KGaA) containing halt protease and phosphatase inhibitor cocktail (100X) (Catalog number 78440, Thermo Fisher Scientific). Nuclear extracts were obtained by NE-PER Nuclear and Cytoplasmic Extraction Reagents kit (Catalog number 78833, Thermo Fisher Scientific), according to the manufacturer’s protocol. Protein concentration was assessed by BCA assay kit (Catalog number 23227, Thermo Fisher Scientific). The extracts were either immediately used or stored at −80°C until further use.

**Western Blotting**

Equal amounts of protein samples (30μg of proteins per lane) were diluted in the sample buffer and then loaded and resolved onto 10 or 15% Bolt Bis-Tris plus mini gels. Next, the iBlot 2 gel transfer stacks mini integrated with nitrocellulose transfer membranes were used to transfer protein by using the dry blotting iBlot 2 gel transfer device (all provided by Invitrogen-Thermo Fisher Scientific). The membranes were blocked with 5% non-fat dry milk for 30 minutes, then incubated with primary antibodies overnight at 4°C and incubated with appropriate secondary antibodies. The antibodies used are listed in supplementary material, **Table S1**. Detection was achieved using HRP-conjugated anti-rabbit and anti-mouse antibodies. Immunoreactive bands were detected by enhanced chemiluminescence with clarity Western ECL substrate (Bio-Rad Laboratories Inc., Hercules, CA, USA) and images were captured by iBright imaging systems (Invitrogen-Thermo Fisher Scientific). Protein expression was quantified by densitometric analysis by using ImageJ software, version 1.8.0 (National Institutes of Health).

**RT-qPCR in HB cells**

Total RNA extraction from cell line was performed using Total RNA Purification Plus Kit (Catalog number 48300, Norgen Biotek Corp., Thorold, ON, Canada), according to the manufacturer’s protocol. Next, the protocol was the same of that described for liver tissue samples. The mRNA level expression of target genes was determined by using specific TaqMan commercial probes by Applied Biosystems-Thermo Fisher Scientific: these included *AFP* (AFP Hs01040598_m1), *EPCAM* (EPCAM Hs00901885_m1), *OCT4* (POU5F1 Hs01654807_s1), *SOX2* (SOX2 Hs01053049_s1). The mRNA levels were normalized to endogenous control gene eukaryotic 18S rRNA (18S Hs99999901_s1). Based on the ΔΔCt method, relative amounts of mRNA were expressed as fold changes versus control.

# Supplementary Figures and Tables

**Supplementary Table 1.** List of antibodies.

| Protein target | Dilution | Source | Catalogue Number |
| --- | --- | --- | --- |
| *FAK* | 1:1000 (WB)  1:200 (IF) | Cell Signaling Technology | 13009 |
| *pTyr397FAK* | 1:1000 (WB) | Cell Signaling Technology | 8556 |
| *pTyr397FAK* | 1:100 (IF) | Thermo Fisher Scientific-Invitrogen | 700255 |
| *EZH2* | 1:1000 (WB)  1:100 (IF) | BD Biosciences | 612666 |
| *HDAC2* | 1:1000 (WB)  1:200 (IF) | Cell Signaling Technology | 5113 |
| *H3K27me3* | 1:700 (WB)  1:300 (IF) | Cell Signaling Technology | C36B11 |
| *H3K27ac* | 1:700 (WB)  1:300 (IF) | Cell Signaling Technology | PA596618 |
| *β-Catenin* | 1:1000 (WB) | Santa Cruz Biotechnology | sc-7963 |
| *β-Catenin* | 1:100 (IHC) | Leica Biosystems | PA0083 |
| *α-Tubulin* | 1:5000 (WB) | Novus Biologicals | NB100-690 |
| *Histone H3* | 1:1000 (WB) | Cell Signaling Technology | 4499 |
| *HRP-conjugated anti-rabbit* | 1:7000 (WB) | Jackson ImmunoResearch | 111-035-144 |
| *HRP-conjugated anti-mouse* | 1:7000 (WB) | Jackson ImmunoResearch | 115-035-146 |
| *Rabbit IgG (H+L) Cross-Adsorbed Secondary Antibody Alexa Fluor 488* | 1:500 (IF) | Thermo Fisher Scientific-Invitrogen | A-11070 |
| *Mouse anti-Human IgG1 Fc Secondary Antibody Alexa Fluor 488* | 1:500 (IF) | Thermo Fisher Scientific-Invitrogen | A-10631 |

**
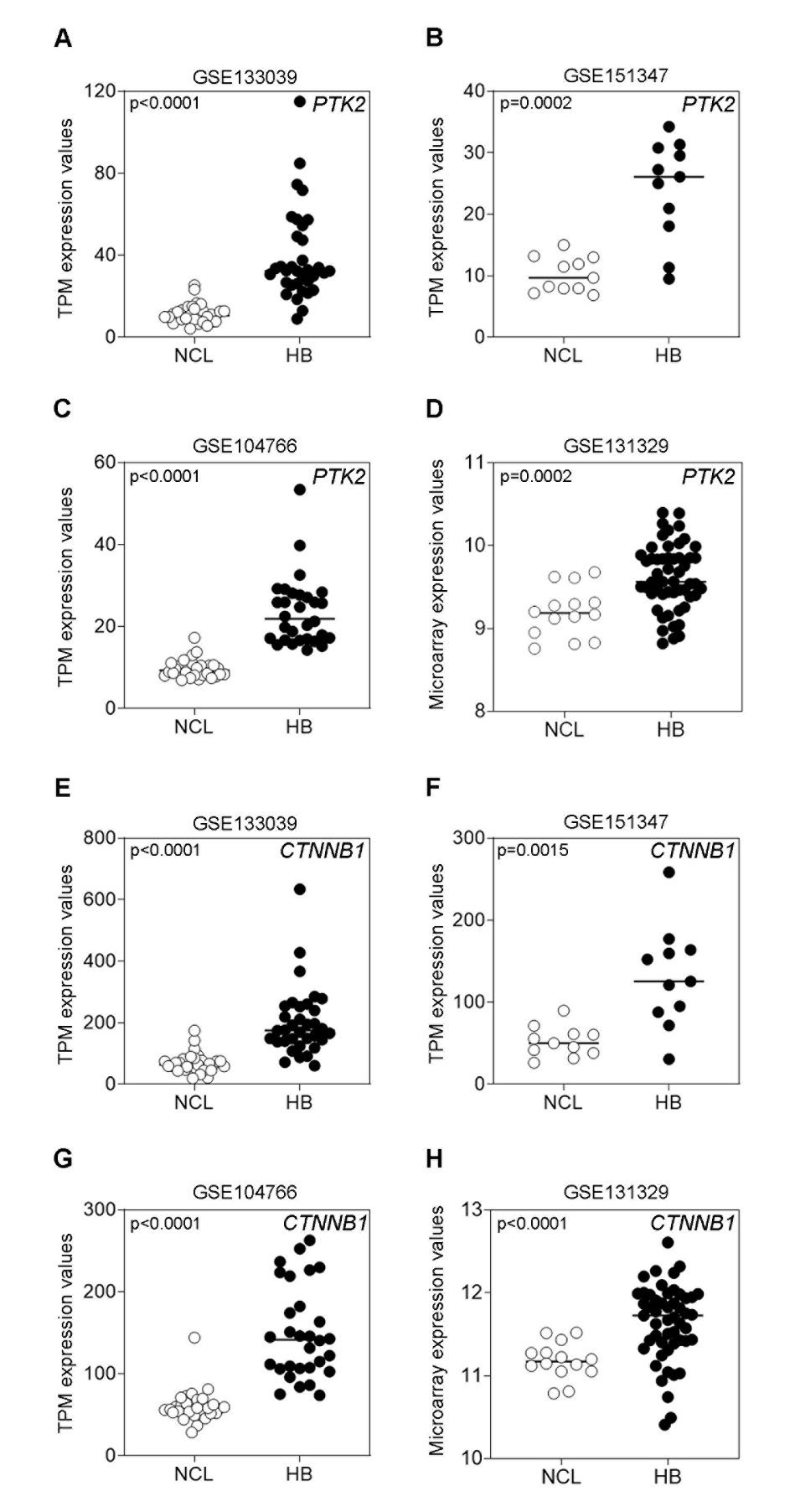
**

**Supplementary Figure 1. FAK and β-Cat gene expression in HB tissues and in noncancerous liver (NCL).** TPM expression values of *PTK2* **(A)** and *CTNNB1* **(E)** genes in the GSE133039 RNA-sequencing dataset in NCL (n=32) and in HB (n=34) tissues; TPM expression values of *PTK2* **(B)** and *CTNNB1* **(F)** genes in the GSE151347 RNA-sequencing dataset in NCL (n=11) and in HB (n=11) tissues; TPM expression values of *PTK2* **(C)** and *CTNNB1* **(G)** genes in the GSE104766 RNA-sequencing dataset in NCL (n=30) and in HB (n=30) tissues; microarray expression values of *PTK2* **(D)** and *CTNNB1* **(H)** genes in the GSE131329 microarray dataset in NCL (n=14) and in HB (n=53) tissues.

**Supplementary Table 2.** Patient characteristics and clinical features.

|  | Sex | Age at diagnosis (months) | Nuclear  β-Cat | GPC3 | GS | AFP levels  (ng/mL) | Tumor size  (cm) | Metastasis | Histology  subtype | Surgery | Therapy | Outcome |
| --- | --- | --- | --- | --- | --- | --- | --- | --- | --- | --- | --- | --- |
| HB1 | M | 20 | 1 | 3 | 1 | 137380 | 8 | NO | Mixed | RH | SIOPEL 3HR | Alive |
| HB2 | F | 14 | 1 | 2 | 1 | 37406 | 2.5 | NO | Mixed | RH | - | Alive |
| HB3 | M | 6 | 0 | 1 | 1 | 126854 | 11 | YES, lung | Embryonal | RH | - | Dead |
| HB4 | F | 20 | 2 | 2 | 1 | 677 | 5 | NO | Fetal | LT | - | Alive |
| HB5 | M | 42 | 2 | 0 | 1 | 121000 | 7 | YES, lung | Mixed | RH | SIOPEL 4 | Alive |
| HB6 | M | 17 | 1 | 3 | 1 | 3157 | 5 | NO | Fetal | RH | Cisplatin | Alive |
| HB7 | F | 39 | 1 | 2 | 1 | 192889 | 4 | NO | Mixed | RH | SIOPEL 3 | Alive |
| HB8 | F | 29 | 1 | 3 | 1 | 674122 | 7 | NO | Mixed | LH | SIOPEL 4 | Alive |
| HB9 | F | 2 | 2 | 1 | 1 | 19777 | 3.5 | NO | Embryonal | LH | SIOPEL 4 | Alive |
| HB10 | F | 21 | 2 | 3 | 1 | 595794 | 4.8 | YES, lung | Mixed | LH | SIOPEL 4 | Alive |
| HB11 | M | 127 | 2 | 3 | 1 | 78538 | 6.5 | YES, lung | HCN NOS | RH | SIOPEL 4 | Alive |
| HB12 | M | 12 | 2 | 0 | 0 | 3394 | 3 | NO | Mixed | RH | SIOPEL 6 | Alive |
| HB13 | M | 14 | 0 | 0 | 0 | 1771 | 2 | NO | Embryonal | LH | SIOPEL 4 | Alive |
| HB14 | M | 19 | 0 | 0 | 0 | 6942 | 1.3 and 4 | NO | Mixed | LH | SIOPEL 3 | Alive |
| HB15 | F | 7 | 1 | 1 | 1 | 856000 | 7 | NO | Fetal | LH | SIOPEL 3 | Alive |
| HB16 | M | 43 | 0 | 1 | 1 | 35930 | 8.5 | NO | HCN NOS | RH | Cisplatin/doxorubicin | Alive |
| HB17 | F | 16 | 1 | 1 | 1 | 141655 | 6 | NO | Mixed | RH | SIOPEL 3 | Alive |

Abbreviations: AFP, alpha-fetoprotein; β-Cat, β-Catenin; GPC3, Glypican3; GS, Glutamine Synthetase; HCN NOS, hepatocellular neoplasm-not otherwise specified; RH, right hepatectomy; LF, left hepatectomy. Mixed definition includes epithelial-mesenchymal histotype.

**
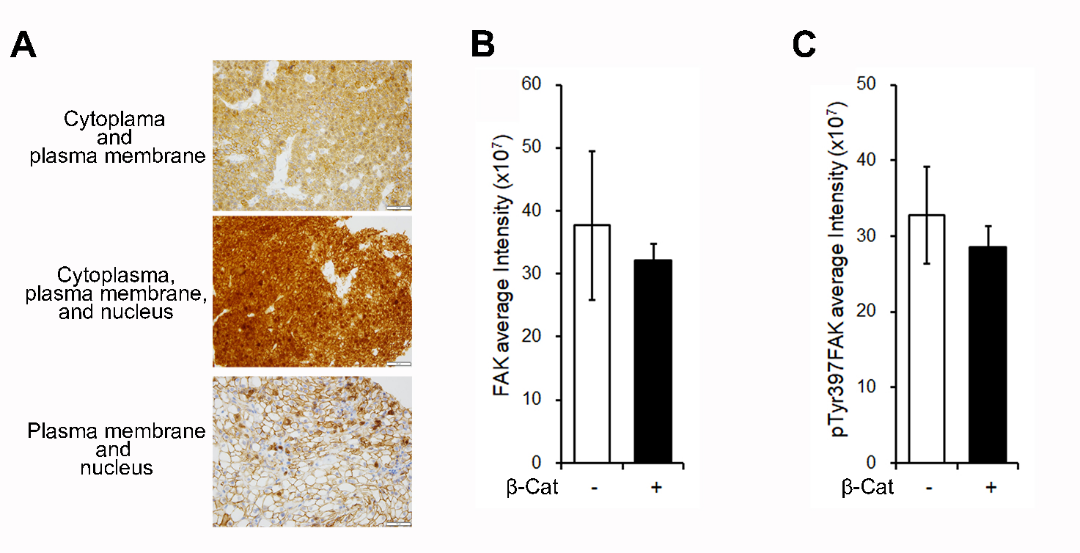
**

**Supplementary Figure 2. Correlations between FAK, pTyr397FAK and nuclear β-Cat in HB tissues.** **(A)** Representative images of β-Cat immunohistochemical staining in HB tissues. 40X magnification; **(B)** correlation between average fluorescence intensity for total FAK and the presence (+) or absence (-) of β-Cat in the nucleus in HB (n=17) tissues; and **(C)** correlation between average fluorescence intensity for pTyr397FAK and the presence (+) or absence (-) of β-Cat in the nucleus in HB (n=17) tissues.

**
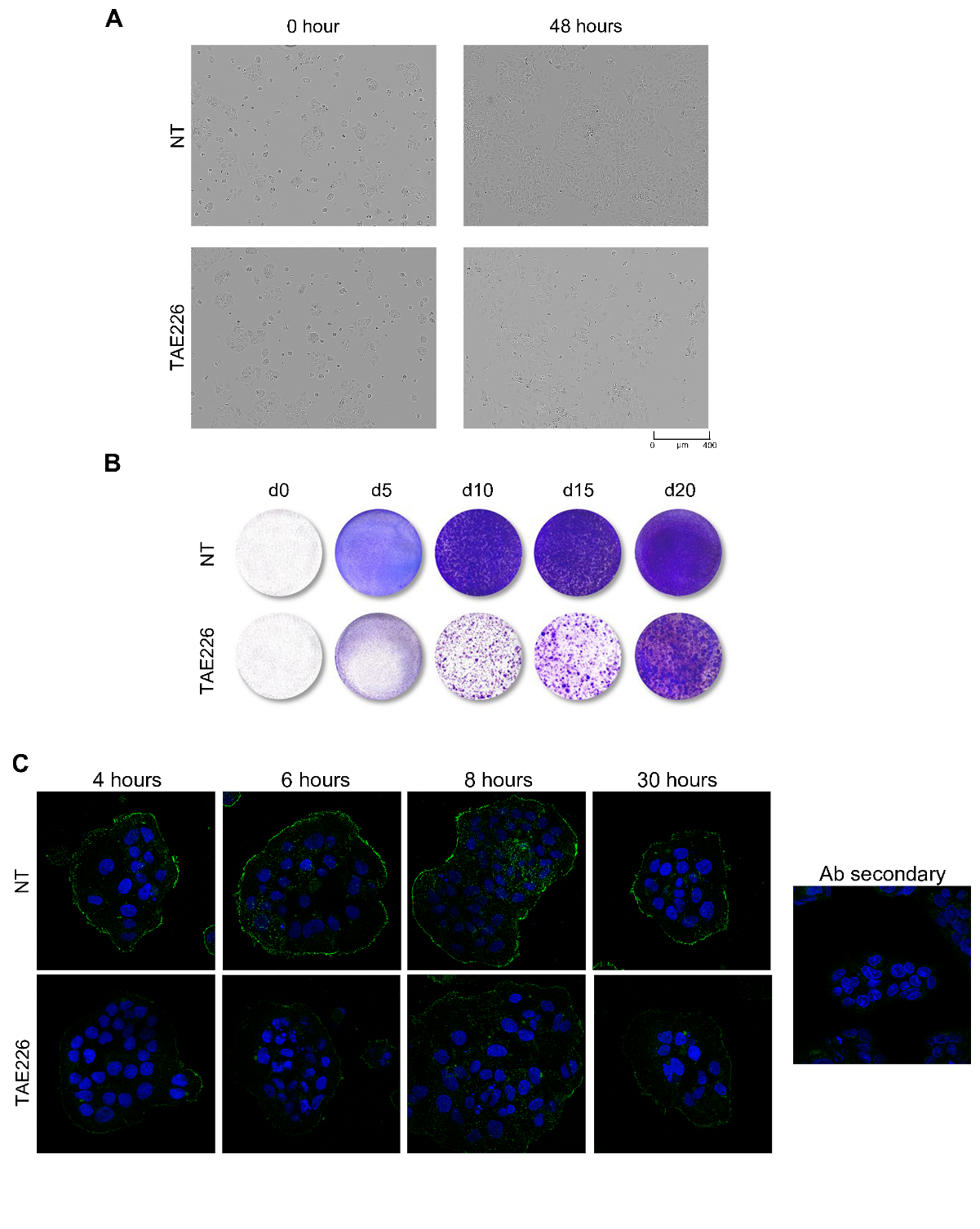
**

**Supplementary Figure 3. The FAK inhibitor TAE226 reduces growth of Huh6 human HB cells. (A)** Representative contrast phase images of cell confluence at initial time (0 hour) and after 48 hours in Huh6 cells non-treated (NT) or treated with 7µM TAE226 (TAE226). 10X magnification; **(B)** representative images showing colonies produced after 0, 5, 10, 15 or 20 days (d) of incubation by Huh6 cells NT or treated with TAE226; **(C)** representative immunofluorescence by confocal imaging of pTyr397FAK (green) in Huh6 cells NT or TAE226 at different timepoints. 60X magnification. Hoechst nuclear staining (blue).

**
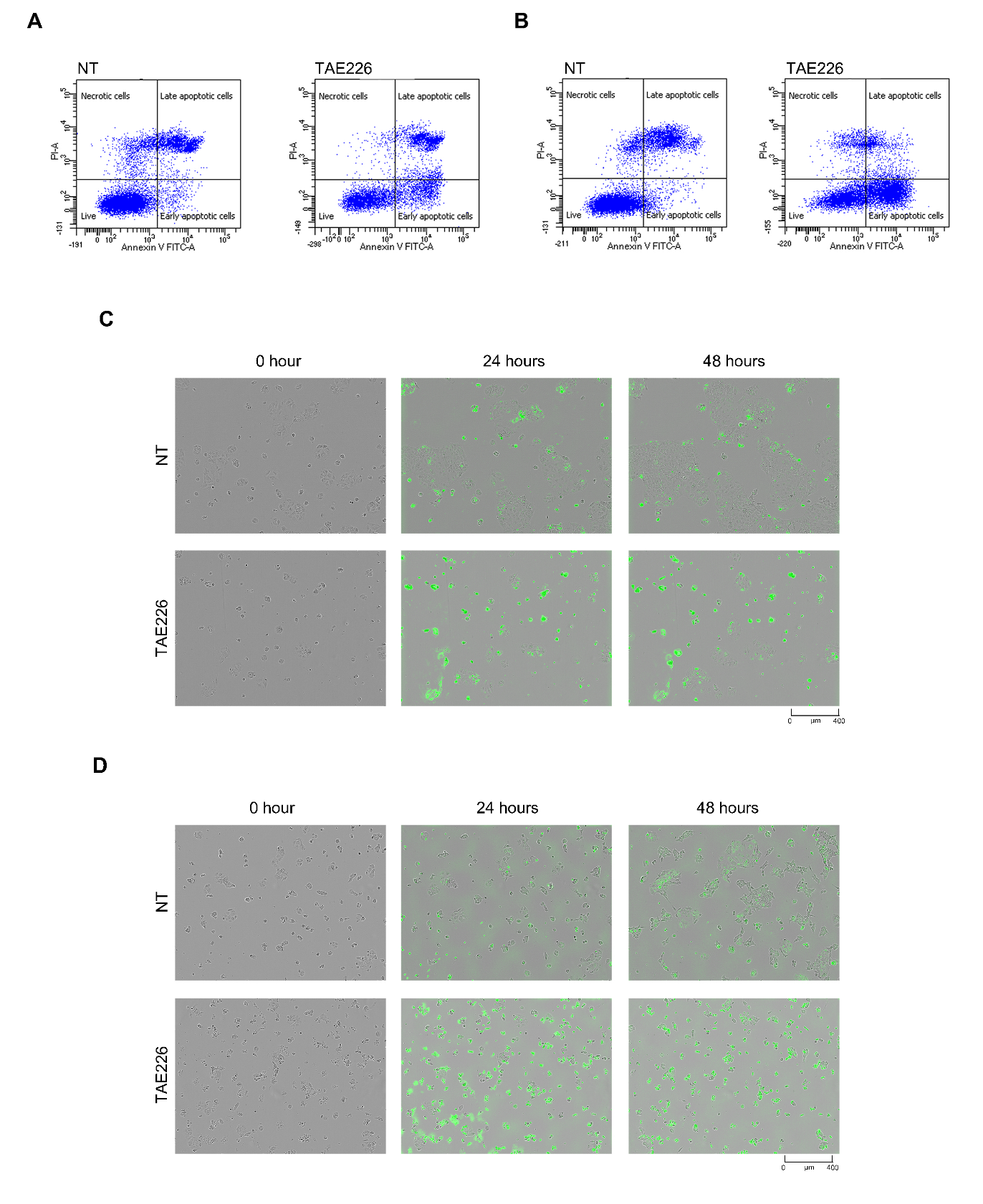
**

**Supplementary Figure 4. The FAK inhibitor TAE226 induces apoptosis in Huh6 human HB cells.** Representative histograms of Annexin V-FITC and propidium iodide staining and sorting by flow cytometry in Huh6 cells NT or treated with TAE226 for 24 hours **(A)** and 48 hours **(B)**. Cells in the quadrants represent necrotic, late apoptotic, live and early apoptotic populations, respectively. Representative images of Huh6 cells NT or treated with TAE226 stained with Annexin V **(C)** and Caspase 3/7 **(D)** after 0, 24, and 48 hours of incubation.
